# *Brachyspira hyodysenteriae* detected in a dog with acute haemorrhagic diarrhoea: a case report

**DOI:** 10.1186/s12917-026-05764-6

**Published:** 2026-08-04

**Authors:** Hyeji Jeong, Hyojin Ahn, Doo-sung Cheon, Yein Oh, Won-Kyoung Yoon

**Affiliations:** 1 Department of Veterinary Internal Medicine, College of Veterinary Medicine and Institute for Veterinary Biomedical Science, Kyungpook National University, Daegu, 41566 Republic of Korea; 2 New S Animal Hospital, Gyeonggi-do, 15865 Republic of Korea; 3 Postbio Inc, Gyeonggi-do, 12106 Republic of Korea; 4 Guardian Angel Veterinary Hospital, Gyeonggi-do, 13988 Republic of Korea

**Keywords:** Diarrhoea, Swine dysentery, *Brachyspira hyodysenteriae*, Dog, PCR

## Abstract

**Background:**

*Brachyspira hyodysenteriae* is a well-known pathogen of swine dysentery, a severe mucohaemorrhagic colitis in pigs. While several *Brachyspira* species are reported in dogs, natural *Brachyspira hyodysenteriae* detection in dogs is extremely rare. This case report aims to provide insight into the pathogenic potential and clinical significance of *Brachyspira hyodysenteriae* in dogs.

**Case presentation:**

A 5-year-old male mongrel dog was presented with acute haemorrhagic diarrhoea shortly after being adopted from a shelter, where he had a history of soft stools. The faeces were watery, containing fresh blood and fibrinous mucus. Initial wet mount and faecal flotation tests were negative for parasites or ova, but faecal smear cytology revealed diverse bacterial morphologies, including chevron- and spiral-shaped forms. Real-time PCR identified *Brachyspira hyodysenteriae* with a low Ct value (27.19), alongside *Campylobacter coli*, *Campylobacter jejuni*, canine astrovirus and *Cystoisospora*. The dog was treated with enrofloxacin, metronidazole, and fenbendazole, leading to complete resolution of haematochezia and gradual normalization of stool consistency. Follow-up real-time PCR confirmed the absence of all previously detected pathogens, and the patient remained asymptomatic.

**Conclusions:**

This case suggests that *Brachyspira hyodysenteriae*, traditionally regarded as a swine-specific pathogen, may contribute to gastrointestinal disease in dogs. However, because confirmatory bacterial culture was not performed and multiple enteropathogens were detected concurrently, a definitive association between *Brachyspira hyodysenteriae* and the observed haemorrhagic diarrhoea cannot be established. Nevertheless, it highlights the importance of considering *Brachyspira hyodysenteriae* in unexplained cases of infectious diarrhoea in dogs, particularly regarding mixed infections. These findings also raise concerns regarding potential interspecies transmission and associated biosecurity risks. Further research is warranted to enhance understanding of diagnostic strategies, treatment approaches and antibiotic resistance patterns.

## Background

*Brachyspira* is an anaerobic, Gram-negative spirochete distinguished based on its high motility and species-dependent β-haemolytic activity. The genus currently comprises nine formally recognised species, along with several provisional ones, and is commonly detected in the intestinal tracts of many animals, including birds and mammals. While certain species are pathogenic and associated with gastrointestinal diseases, including diarrhoea, others appear to be non-pathogenic and may act as commensals [1].

In dogs, *B. canis* is regarded as a non-pathogenic commensal, whereas *B. pilosicoli* has been identified in dogs with diarrhoea [2]. However, *B. pilosicoli* has also been detected in clinically healthy dogs using antigen- and antibody-based assays [3, 4], and its pathogenic role in dogs remains controversial. In a recent epidemiological cohort study, *Brachyspira* spp. were isolated from approximately 11% of dogs, with most isolates identified as *B. canis* [4]. These findings suggest that, although *B. pilosicoli* may act as a potential pathogen, *Brachyspira* detection in dogs more commonly reflects commensal colonization than clinically significant infection. Similarly, in swine, species such as *B. innocens* are regarded as non-pathogenic commensals, whereas *B. hyodysenteriae*, *B. hampsonii* and *B. suanatina* are recognised enteric pathogens associated with diarrhoea. Among these, *B. hyodysenteriae* is the principal etiologic agent of swine dysentery, a severe fibrinous, mucohaemorrhagic diarrhoeal disease [1]. In susceptible pigs, *B. hyodysenteriae* penetrates the colonic mucus layer, establishes mucosal colonisation, and induces epithelial injury, leading to malabsorptive diarrhoea [1]. Although the precise processes of tissue destruction are incompletely understood, hemolysins and lipooligosaccharide are thought to contribute to epithelial sloughing and secondary bacterial invasion [1]. However, the relevance of these mechanisms to canine enteric disease remains poorly defined.

Several studies in small animal veterinary medicine have focused on investigating the role of *B. pilosicoli* in infectious diarrhoea in dogs, partly because of its zoonotic potential [5]. Conversely, reports of *B. hyodysenteriae* detection in dogs are extremely rare, and it is not considered to be zoonotic [1]. According to one review, the report by Songer et al. remains the only peer-reviewed, English-language publication describing the presumed detection of *B. hyodysenteriae* in a dog residing on a pig farm [6, 7]. Nonetheless, this case was reported before non-pathogenic *B. innocens* was taxonomically distinguished from *B. hyodysenteriae*, which should be considered when interpreting the findings [8]. From a clinical perspective, dogs presenting with diarrhoea are typically evaluated for common enteropathogens such as *Giardia* [9], whereas *Brachyspira* is not routinely included in standard diagnostic panels and may therefore be overlooked. Awareness of this pathogen may be important for improving appropriate diagnostic testing. Here, we present a rare case in which *B. hyodysenteriae* was identified in a dog with diarrhoea, alongside *Campylobacter jejuni*, *Campylobacter coli*, astrovirus and *Cystoisospora*.

## Case presentation

**Fig. 1:**
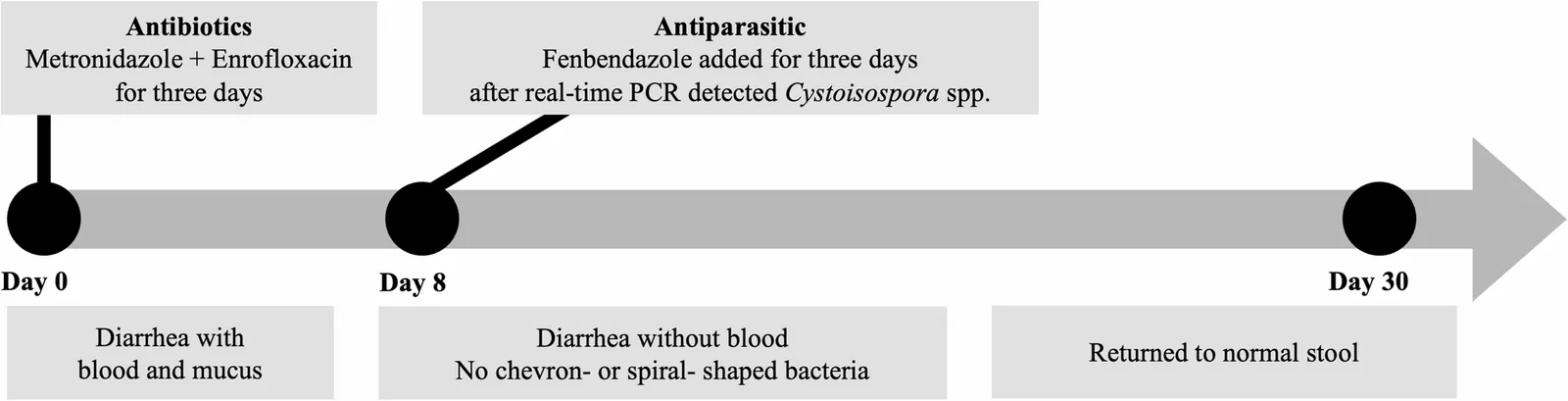
Timeline of treatment interventions and clinical outcomes in the patient. The dog presented with acute haemorrhagic diarrhoea and was treated with metronidazole, enrofloxacin, and fenbendazole following the diagnosis of a mixed infection, including *Brachyspira hyodysenteriae.* Progressive clinical improvement was observed over the subsequent weeks

A 5-year-old male mongrel dog was presented with acute haemorrhagic diarrhoea, while appetite and vital parameters remained normal (day 0; Fig. 1). The dog had been adopted from a shelter the previous day and reportedly had a history of soft stools while there. On physical examination, he appeared clinically stable, with a rectal temperature of 38.6 °C and a body condition score of 5/9. Dehydration was estimated at < 5%, based on the history of diarrhoea. Haematologic analysis, including complete blood count and serum biochemistry results, revealed no significant abnormalities.

**Fig. 2:**
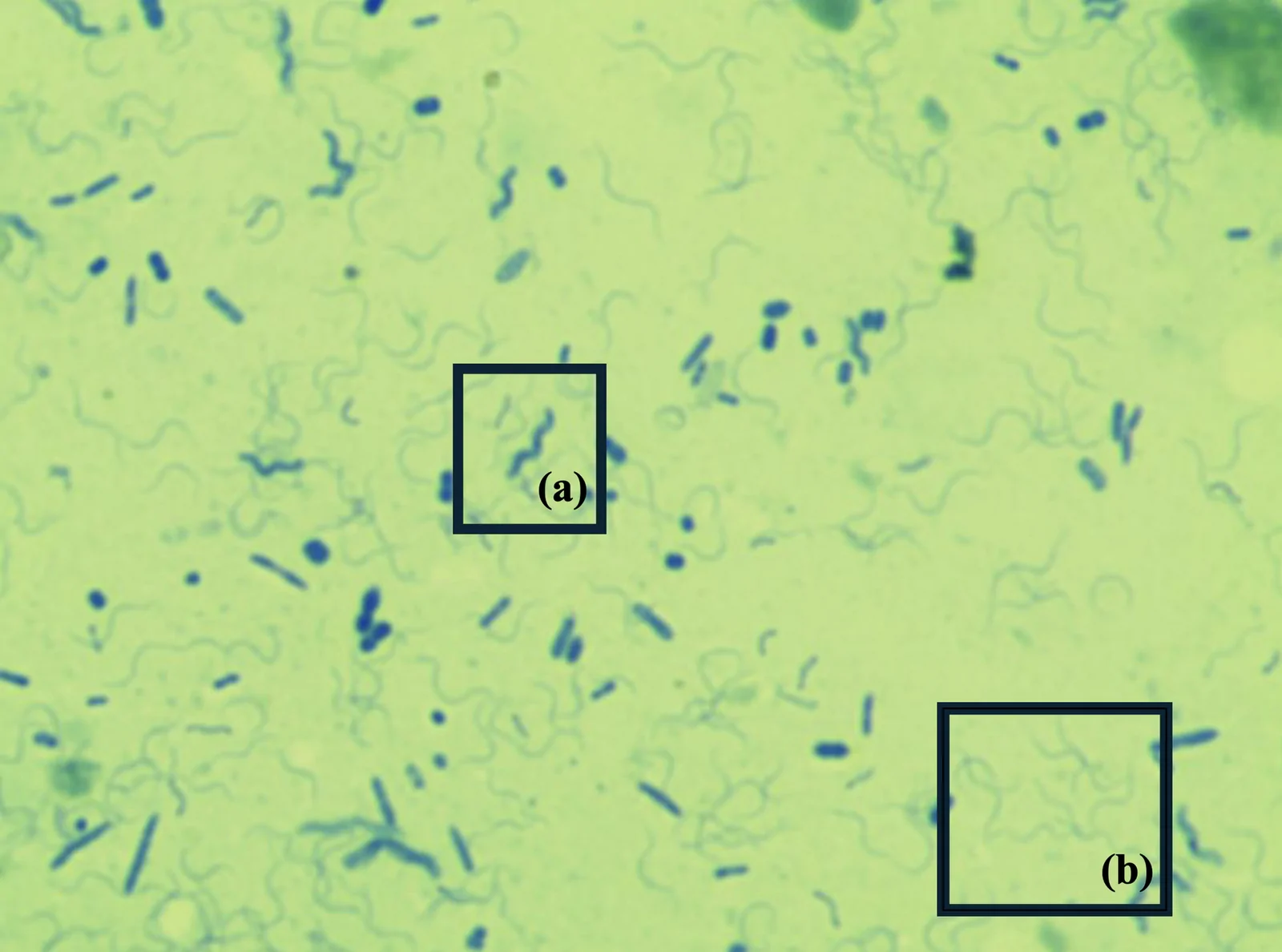
Faecal smear cytology of the stool sample on day 0. Diff-Quik stain, ×1000 (oil immersion). Note the chevron-shaped bacteria (**a**) and the spiral-shaped bacteria (**b**)

According to the owner, the faeces were completely liquid in consistency and contained fresh blood and mucus with a fibrinous texture, suggestive of colonic diarrhoea. Faecal wet mount and flotation test revealed no parasites or ova. However, faecal smear cytology revealed abundant bacteria, including cocci, rods, chevron-shaped bacteria, and spiral-shaped bacteria (Fig. 2). Subsequently, faecal samples were collected directly from the rectum, placed in universal transport medium, and submitted to a commercial veterinary diagnostic laboratory (Pobanilab, Republic of Korea) for real-time PCR analysis of enteric pathogens. In additional to the standard gastrointestinal pathogen panel—including canine distemper, canine parvovirus, canine enteric coronavirus, canine norovirus, canine circovirus, canine sapovirus, canine astrovirus, group A rotavirus, *Clostridium perfringens*, *Campylobacter jejuni*, *Campylobacter coli*, *Salmonella* spp., enteropathogenic *Escherichia coli*, enterotoxigenic *Escherichia coli*, enterohemorrhagic *Escherichia coli*, enteroinvasive *Escherichia coli*, *Cryptosporidium parvum*, *Giardia lamblia*, *Entamoeba histolytica*, *Cystoisospora* spp., *Lawsonia intracellularis*, *Toxocara* spp., *Toxoplasma gondii*, *Ancylostoma* spp. and *Necator americanus*—testing for spirochetes, including *Brachyspira* spp., was also performed.

As a preventive measure, the patient received an oral dose of Drontal Plus^®^ (Bayer, Germany)—a dewormer containing febantel, pyrantel pamoate, and praziquantel—as one tablet, despite the absence of detectable parasites or ova in clinical examination. Additionally, the patient received an intra-muscular injection of Kingberine^®^ (Samyang Anipharm Co., Ltd., Republic of Korea)—an astringent–antibiotic combination containing berberine chloride, sulfamethoxazole sodium, and sulfadimethoxine sodium—at a dose of 0.1 mL/kg. At discharge, the patient was prescribed oral metronidazole (15 mg/kg BID; HK inno.N Corp., Republic of Korea), enrofloxacin (5 mg/kg SID; Ashish Life Science Pvt. Ltd., India), and famotidine (0.5 mg/kg BID; Nelsonpharm, Republic of Korea) for 3 days (day 0–3).

On day 3, the results of the real-time PCR test were received, revealing the presence of *B. hyodysenteriae* with the lowest Ct value of 27.19. Additionally, *Campylobacter coli*, *Campylobacter jejuni*, canine astrovirus and *Cystoisospora* were also detected (Table 1). On the same day, haematochezia had completely resolved, and only soft stools remained; therefore, antibiotic therapy was discontinued. Repeat faecal smear cytology also showed no chevron- or spiral-shaped bacteria, supporting the decision to discontinue further antibiotics. Unexpectedly, the real-time PCR test detected *Cystoisospora*, a parasite that had not been previously identified in the clinic on day 0. Consequently, complete resolution of the infection could not be confirmed. Therefore, from days 8–11, fenbendazole (Panacur^®^, Intervet Inc) was administered for an additional 3 days at 50 mg/kg SID. Faecal consistency continued to improve, and on day 30, normal stool consistency was fully restored (Fig. 1).

A follow-up real-time PCR test on day 30 showed no detectable levels of any previously identified pathogens (Table 1). The patient remained asymptomatic, with no recurrence of diarrhoea.

**Table 1:** Real-time PCR result of the stool specimens on days 0 and 30

Pathogen detected by real-time PCR	Day 0 (Ct value)	Day 30 (Ct value)
*Brachyspira hyodysenteriae*	27.19	Undetected
*Campylobacter coli*	30.77	Undetected
*Campylobacter jejuni*	31.14	Undetected
Canine astrovirus	33.07	Undetected

## Discussion and conclusions

In this case, the patient presented with haemorrhagic diarrhoea, and *B. hyodysenteriae* was detected alongside *Campylobacter jejuni*, *Campylobacter coli*, astrovirus and *Cystoisospora*. *B. hyodysenteriae* is a well-recognised pathogen in pigs, but reports of its natural detection in dogs are extremely rare, with only a single peer-reviewed case report in English documented to date [6, 7]. Nevertheless, the present findings raise the possibility that *B. hyodysenteriae* may have contributed to the gastrointestinal disease observed in this dog.

*B. hyodysenteriae* causes swine dysentery, a disease characterised based on colonic mucosal destruction, fibrinous mucohaemorrhagic diarrhoea, growth retardation, and, in severe cases, death in post-weaning pigs [1]. In this case, the patient presented with fibrinous mucohaemorrhagic colonic diarrhoea, a low Ct value for *B. hyodysenteriae* on real-time PCR, and showed rapid clinical improvement following antibiotic treatment. These findings raise the possibility that *B. hyodysenteriae* contributed to the clinical presentation rather than representing an incidental co-detected organism. Notably, while the clinical presentation resembled swine dysentery, the absence of systemic signs suggests that the disease may present in a milder form in dogs.

However, the simultaneous detection of multiple pathogens, including *Campylobacter jejuni*, *Campylobacter coli*, astrovirus, and *Cystoisospora*, complicates the interpretation of the clinical signs. Although *Campylobacter* spp. are frequently isolated from healthy dogs and asymptomatic carriage is common, they have also been associated with acute and haemorrhagic diarrohea, particularly in young or stressed animals [10]. Likewise, astrovirus and *Cystoisospora* are generally associated with mild small intestinal diarrhoea; however, *Cystoisospora* has been shown to amplify its pathogenicity during co-infection, especially in immunocompromised patients, leading to haemorrhagic diarrhoea [11]. These observations raise the possibility that concurrent enteropathogens may have interacted with one another, thereby influencing clinical manifestation of disease in the present case. Consistent with this concept, a recent experimental study on pigs demonstrates that co-infection with *B. hyodysenteriae* and *Lawsonia intracellularis* results in more severe clinical signs, lesions and bacterial loads than that of individual infections [12]. Taken together, the concurrent detection of multiple enteropathogens precludes definitive attribution of the haemorrhagic diarrhoea to any single organism. Nevertheless, the possibility that co-infecting enteropathogens contributed to or enhanced the pathogenic expression of *B. hyodysenteriae*, as demonstrated in experimental co-infection models, warrants further investigation.

Additionally, several studies report that changes in gut microbiome composition and dietary intake can influence the expression of *B. hyodysenteriae*, with its pathogenicity being highly dependent on the presence of specific mucolytic bacteria. In pigs, the ingestion of highly fermentable fibre promotes the growth of beneficial commensal bacteria, suppresses pathogenic mucolytic bacteria, and consequently helps prevent swine dysentery [13]. Despite differences in digestive physiology, it remains possible that host factors—including gut microbiome composition, nutritional status and mucosal immune response—may have influenced the manifestation of disease in this case.

Previous studies suggest that *B. hyodysenteriae* detected in diarrheic dogs on pig farms may reflect transient colonisation or true infection, often associated with ingestion of faces from pigs affected via swine dysentery [7]. The patient in this case had no history of faecal ingestion or contact with pig farms. However, because the dog was recently adopted from a shelter, prior exposure cannot be completely ruled out, and the source of acquisition remains unknown. In humans, *B. hyodysenteriae* does not colonise the intestinal tract or cause diseases, indicating clear barriers to cross-species infection [14]. Nevertheless, the possibility of limited interspecies transmission—particularly to dogs in this context—cannot be excluded. Further epidemiological studies are needed to clarify whether non-porcine or indirect reservoirs of *B. hyodysenteriae* exist.

In this case, *B. hyodysenteriae* were identified through microscopic examination and species-specific real-time PCR, a widely used and reliable approach for differentiating *Brachyspira* spp [15]. While conventional PCR protocols for *Brachyspira* spp. typically employ genus-specific primers followed by downstream analyses such as deep sequencing to achieve species differentiation [16], species-targeted PCR enables direct identification of individual *Brachyspira* species.

While additional diagnostic tools—such as selective anaerobic culture, phenotypic (biochemical and haemolytic) characterisation of isolates, and histopathologic evaluation—could have provided further confirmation, they were not employed and represent a limitation of this case. Nonetheless, PCR remains a valuable method for *Brachyspira* spp. detection, despite reported concerns about its specificity. For example, one study reports false-positive rates as high as 80% in *B. hampsonii-* and *B. intermedia*-specific PCR assays [17]. These findings underscore the need for cautious interpretation of PCR results and highlight the complementary value of culture-based methods, which not only provide microbiological confirmation but also facilitate phenotypic characterization, thereby providing additional evidence regarding the pathogenic potential.

Pleuromutilins—such as tiamulin and valnemulin—are the first-line treatment for *B. hyodysenteriae* in pigs. These antibiotics are widely used in swine across many countries and, in some cases, are also applied in poultry [18]. However, their use in small animal veterinary medicine remains relatively uncommon. In humans, metronidazole is commonly applied to treat intestinal spirochetosis caused by *B. pilosicoli* [19]. In this case, the patient received metronidazole and additional antibiotics, leading to gradual improvement and subsequent disappearance of the bacteria. Nevertheless, since acute diarrhoea in dogs is not typically treated with antibiotics, it remains uncertain whether the observed improvement was attributed to their administration. Given the rising prevalence of antibiotic resistance in *Brachyspira* spp. among swine populations, antibiotic susceptibility testing is recommended prior to treatment to minimise the risk of developing resistance [20].

In conclusion, *B. hyodysenteriae* detection in dogs with haemorrhagic diarrhoea has been very rarely reported to our knowledge. This case supports consideration of *B. hyodysenteriae* as a potential enteropathogen in dogs. However, the absence of culture-based confirmation and the inherent specificity concerns of PCR assays limit the diagnostic certainty, and these findings should be interpreted cautiously. Further studies are warranted to clarify the pathogenic mechanisms of *B. hyodysenteriae* in dogs, evaluate its potential for interspecies transmission, and characterise its antibiotic resistance patterns.

## Data Availability

All data generated or analysed during this study are included in this published article. Additional information is available from the corresponding author upon reasonable request.
